# ERRATUM: “Malaria in Brazil: what happens outside the Amazonian endemic region”

**DOI:** 10.1590/0074-02760140228ER

**Published:** 2018-08-27

In the article “Malaria in Brazil: what happens outside the Amazonian endemic region”, DOI number: 10.1590/0074-0276140228, published in *Mem Inst Oswaldo Cruz*, Rio de Janeiro, Vol. 109(5): 618-633, August 2014, on pages 621-623 part of the text missing:

**Where it reads:**

The region covers approximately 40.25% of the Brazilian territory, hosts 86.6% (~174 million people) of the population and has 91.6% of the country’s Gross National Product (GNP). In contrast, only approximately 0.5% of the malaria cases registered in Brazil are diagnosed and treated outside the Amazonian endemic region (mean of 1,296 cases/year from 2000-2013) and they present a distinct epidemiological profile. This situation is not comparable in all South American countries that encompass parts of the Amazon Forest in their territories. For example, Colombia has reported the second highest annual number of malaria cases in Latin America (14.2% of all malaria) and most cases (i.e., 90% of malaria cases ceptive area (with competent vectors). An autochthonous case occurs in a location where there is a source of infection. As described in the Atlantic Forest section of this article, this category of malaria may occur as a zoonosis, involving non-human reservoirs and competent vectors. A special variety of malaria known as “airport malaria” occurs when infectious mosquitoes from endemic areas are introduced in a new, malaria-free region and feed on the blood of local residents, usually in the airport neighbourhood, causing an outbreak of the disease. We will not address the “airport malaria” in this paper.

**It should read:**

The region covers approximately 40.25% of the Brazilian territory, hosts 86.6% (~174 million people) of the population and has 91.6% of the country’s Gross National Product (GNP). In contrast, only approximately 0.5% of the malaria cases registered in Brazil are diagnosed and treated outside the Amazonian endemic region (mean of 1,296 cases/year from 2000-2013) and they present a distinct epidemiological profile. This situation is not comparable in all South American countries that encompass parts of the Amazon Forest in their territories. For example, Colombia has reported the second highest annual number of malaria cases in Latin America (14.2% of all malaria) and most cases (i.e., 90% of malaria cases in the country) are reported outside the Colombian Amazon (Arévalo-Herrera et al. 2012).

The cases reported in the extra-Amazonian region include imported, introduced and autochthonous malaria (Table II). Here, we will use the classical definition by the WHO (1961). An imported case arises when an infection is contracted outside the area where the individual resides. The introduced case is a secondary case that is directly derived from a known imported case; in other words, it results from the arrival of a parasitised individual in a receptive area (with competent vectors). An autochthonous case occurs in a location where there is a source of infection. As described in the Atlantic Forest section of this article, this category of malaria may occur as a zoonosis, involving non-human reservoirs and competent vectors. A special variety of malaria known as “airport malaria” occurs when infectious mosquitoes from endemic areas are introduced in a new, malaria-free region and feed on the blood of local residents, usually in the airport neighbourhood, causing an outbreak of the disease. We will not address the “airport malaria” in this paper.

